# Wrist Joint Restriction: Impact on Foot Pressure, Center of Gravity, and the Role of the Dominant Hand

**DOI:** 10.3390/jcm14082829

**Published:** 2025-04-19

**Authors:** Leire Cruz Gambero, Gabriel A. Gijón-Noguerón, Salvador Díaz Miguel, Javier Barón-López, Cantero-Téllez Raquel

**Affiliations:** 1 Physiotherapy Department, Faculty of Health Sciences, University of Malaga, IBIMA Plataforma BIONAND Hand Research Group FE-17, C/Arquitecto Francisco Peñalosa (Ampliación Campus Teatinos), 29010 Malaga, Spain; leiregam@uma.es (L.C.G.); cantero@uma.es (C.-T.R.); 2 Department of Nursing and Podiatry, Faculty of Health Science, University of Malaga, C/Arquitecto Francisco Peñalosa (Ampliación Campus Teatinos), 29010 Malaga, Spain; salvadordiazm@uma.es; 3 Department of Public Health and Psiquiatry, Faculty of Medicine, University of Malaga, Blvr Louis Pasteur, 32 (Campus Teatinos), 29010 Malaga, Spain; baron@uma.es

**Keywords:** wrist immobilization, foot pressure, gravity, foot support

## Abstract

**Background**: Wrist immobilization is a common clinical intervention for wrist injuries; however, its repercussions on gait parameters and plantar support have not been extensively investigated. **Objectives**: The purpose of the study was to determine whether wrist immobilization causes alterations in foot pressure and center of gravity, considering hand dominance and visual conditions (eyes open or closed). **Methods**: The research experiment was conducted using the PodoPrint S4 platform. Basic descriptive statistics were calculated to summarize the variables. Additionally, in the mixed linear model (*t*-tests use Satterthwaite’s method) an analysis of variance for repeated measures (ANOVA-RM) was conducted for the determination of the objectives of the study. **Results**: This study included a total of 44 participants (29 females and 15 males), with an average age of 36.5 years (SD = 17.5). Immobilization, independent of eye condition, resulted in significant alterations in antero-posterior oscillation and in a larger plantar support area. In addition, the results suggest that the eye state significantly influences plantar support, independent of limb immobilization or dominance. **Conclusions**: Our findings reveal significant alterations in antero-posterior oscillation and plantar support due to immobilization, suggesting a dynamic interplay between wrist function and lower limb biomechanics.

## 1. Introduction

Wrist injuries, encompassing distal radius fracture (DRF), triangular fibrocartilage complex injury, instability, scaphoid fracture, and various orthopedic conditions, are commonplace in clinical practice [1,2]. Joint restriction constrains the ability to flexibly coordinate the joints and might lead to reduced balance stability when people perform different tasks or activities [3]. To promote wrist stability and facilitate the healing process, healthcare providers often employ wrist immobilization devices, such as casts or splints [4,5,6]. The potential secondary effects of this intervention on other aspects of an individual’s biomechanics and functional mobility have received limited attention in the scientific literature.

One of the most immediate reactions to upper limb immobilization is an altered gait pattern [7] to compensate for the loss of upper limb function and maintain equilibrium [8]. When the wrist is immobilized, there is a shift in the body’s center of mass. To counteract this shift and prevent falls, individuals may adjust their body posture and engage their core and lower limbs more actively. This adaptive response helps in stabilizing the body’s center of mass during walking [9].

Ford MP et al. [10] confirmed that constraining one arm resulted in an adaptation in trunk and inter-limb coordination when walking at higher velocities in healthy adults, but no specific upper limb extremity movement disorders participants have been included, and studies did not evaluate possible changes in plantar pressure distribution when there is immobilization and consequently no use of the upper limb. This potential alteration in statics could cause postural changes during daily activities that do not involve walking or any form of displacement. Arm swing is a natural component of walking that aids in balance and stability [11]. When upper limb function is compromised due to immobilization, there may be a compensatory reduction or modification in arm swing. Steven H. Collins et al. [11] conducted a series of simulations and human subject experiments with the goal of elucidating the role of arm swinging and its underlying mechanisms in human gait.

Other investigations [11,12,13,14] have concluded that normal arm swing indeed leads to a reduced vertical angular momentum when compared to walking without arm swing. These studies emphasized the imperative to identify alterations in plantar support following wrist or hand immobilization and how these changes may impact not only the movement of the upper limbs but also plantar support. This, in turn, can affect gait cadence and balance, potentially causing challenges or modifications in movement patterns during everyday activities.

Despite the presence of various factors that may affect plantar support following wrist or hand injuries requiring immobilization, such as fear of movement, pain, or catastrophizing, the specific influence of immobilization itself on plantar support remains inconclusive, leaving the potential impact on overall functional recovery uncertain.

The first aim of the present study was to analyze the intra-subject variability in antero-posterior oscillation (APO) and plantar pressure parameters in healthy subjects during the bipedal standing test under wrist–hand dominant and non-dominant immobilization, with both open-eye (OE) and closed-eye (CE) conditions. Based on this aim, we hypothesize that (1) wrist immobilization, regardless of hand dominance, will cause significant alterations in antero-posterior oscillation and plantar pressure distribution when compared to the non-immobilized condition, (2) the effects of wrist immobilization on these parameters will vary depending on hand dominance, and (3) visual conditions (eyes open vs. closed) will significantly influence plantar pressure parameters and postural stability, regardless of wrist immobilization or hand dominance.

## 2. Materials and Methods

### 2.1. Study Design and Participants

The study was performed in the Faculty of Health Science of the University of Málaga. The study was approved by the local ethics committee with the number CEUMA: 97-2022-H and was carried out according to the Helsinki Declaration on ethical principles for research involving human subjects [15]. A convenience sample of 44 participants was selected during November 2022 and December 2023. Informed consent was obtained from all participants before their inclusion in the study.

The inclusion criteria were age ≥ 18 years. The exclusion criteria included cognitive or neurological impairments that could interfere with the instructions, any previously diagnosed musculoskeletal disorders, recent acute injuries (<1 year) in any limb, immobilization within the previous 6 months, and balance problems.

### 2.2. Procedure

Participants were recruited through an open call via social media posts to ensure a diverse and representative sample within the study’s eligibility criteria. Volunteers who expressed interest were invited to participate, provided they met the predefined inclusion criteria outlined in the study protocol. Additionally, all participants received detailed information about the study’s objectives and procedures before providing their informed consent.

Firstly, a questionnaire (designed by the authors) was provided to the participants to obtain the demographic variables, such as age, sex, occupation, physical activity, and hand dominance. The platform pressure data were collected by a researcher who was familiar with the instrument and software, and all measurements were taken by the same evaluator to avoid discrepancies.

The baropodometric measurements were carried out using a baropodometric platform called PodoPrint^®^ S4, a reliable tool for evaluating the distribution of plantar pressures in the dynamic study of the barefoot gait of healthy individuals. The ICCs showed moderate to good repeatability (greater than 0.81 and less than 0.93) [16]. The dimensions of this platform are 57 cm × 57 cm × 9 mm, and it has 1600 sensors. The data were sent digitally to a computer and then processed using software that shows the parameters of plantar pressure, center of pressure, and postural sway. All measurements were taken twice to make data more precise and eliminate possible bias. Assessment was performed in a soundproof room with absolute silence and with no visual distractions. Each participant had to take off any ornaments on their wrists or objects that they had in their pockets, and they had to be barefoot (with no socks). A foot positioning template was used to position the feet at an angle of 30°, with a separation of 2 cm between heels. For every measurement, the participant had to be standing, with resting arms along the body and looking straight ahead at a color signal that was previously placed on the wall. In this position, the following data were obtained for each subject: total foot support surface, measured in cm^2^; average foot pressure in static support, measured in gr/cm^2^; body sway length and body sway area, both in static support; and center of pressures on the *X*-axis (COP X) and *Y*-axis (COP Y), which represent the position of the center of foot pressure compared to the theoretical one.

At first, a quiet stance test was performed for each subject with open eyes (OEs), followed by a second measurement with the same procedure but with closed eyes (CEs). Secondly, a posture–stabilometric test was also performed with OEs and CEs. This procedure was repeated in the same order, immobilizing the wrist of the dominant arm and then immobilizing the other wrist. Dominance was determined by asking the patient which hand they use for writing. We immobilized the wrist with a thermoplastic splint that put the wrist in a neutral position [17,18]. Also, the splint did not cover any fingers or elbow. If the participant coughed, sneezed, turned his/her head, talked, or performed a sudden movement, the measurements were repeated to avoid possible bias.

### 2.3. Data Analysis

The data were processed with R version 4.3.2 for statistical study [19]. The validity conditions (sphericity and normality) were checked. Mauchly tests for sphericity and Greenhouse–Geisser and Huynh–Feldt corrections for departure from sphericity. The Shapiro–Wilk test for normality was used. A mixed linear model (*t*-tests use Satterthwaite’s method) and an analysis of variance for repeated measures (ANOVA-RM) were conducted for the determination of the objectives of the study. Post hoc comparisons with Holm methods were used. For all statistical analyses, the significance level was set at 5%.

## 3. Results

**Figure 1:**
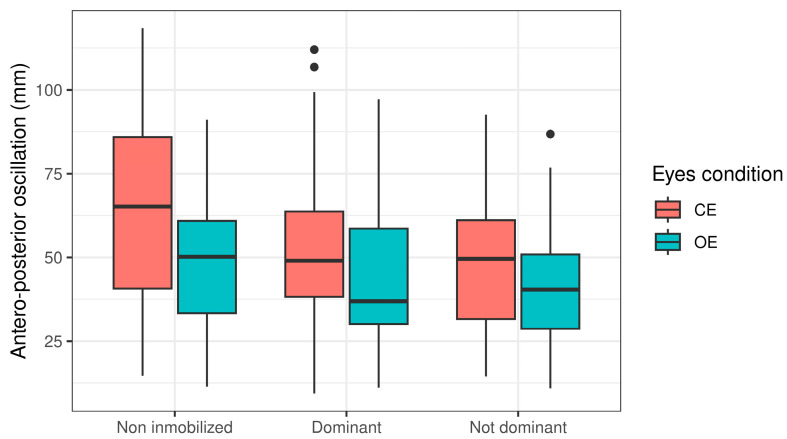
Antero-posterior oscillation associated with state of eyes (open vs. closed).

This study included a total of 44 participants, composed of 29 females and 15 males, with an average age of 36.5 years (SD = 17.5). An initial exploration of the data revealed that the antero-posterior oscillation (APO) varies significantly with the state of the eyes (open vs. closed), as illustrated in Figure 1.

Notably, immobilization alone, independent of eye condition, resulted in significant alterations in APO (*p* < 0.001), as detailed in Table 1.

**Figure 2:**
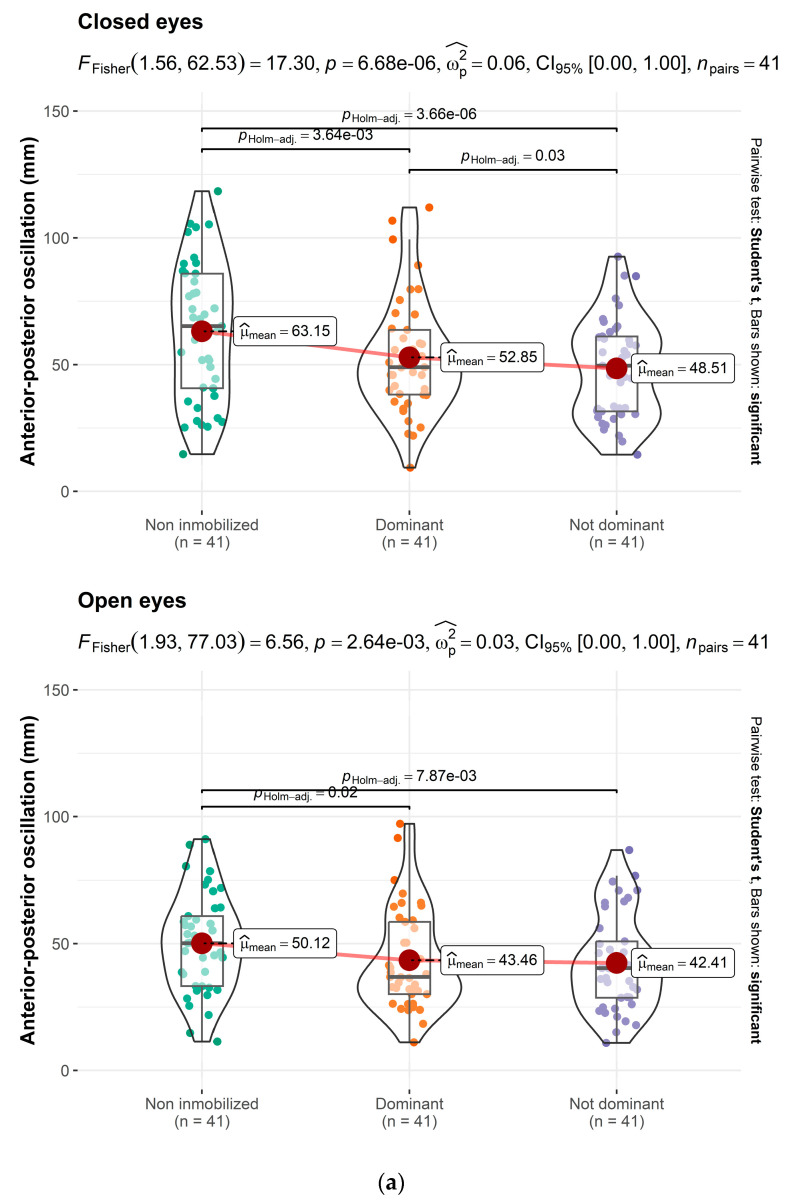

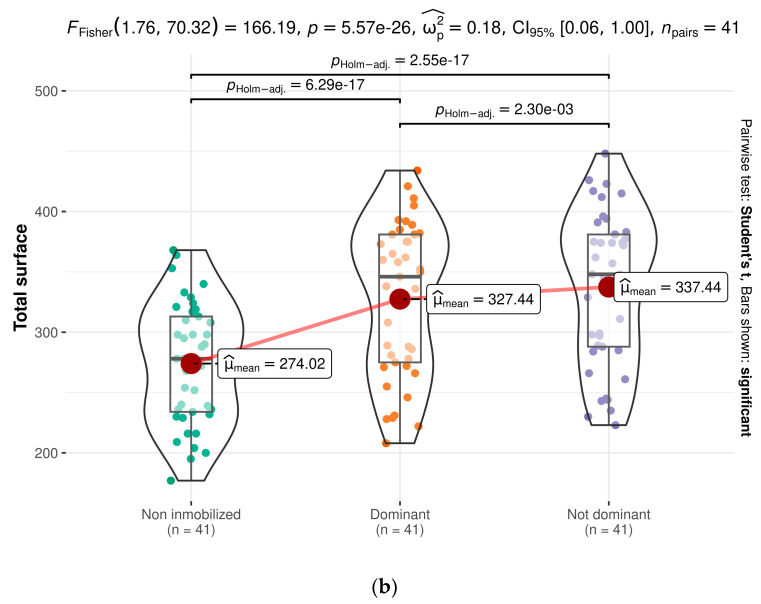
(**a**) Immobilization impacts APO irrespective of eye condition. (**b**). Differences between dominant and non-dominant sides.

Further analysis using an interaction model to assess the effects of eye condition (open vs. closed) separately revealed that immobilization impacts APO irrespective of eye condition (*p* < 0.001), without significant differences between immobilization of the dominant or non-dominant limb (Figure 2a).

Table 2 presents the effects of wrist and hand immobilization on the total plantar support area. The findings indicate a larger plantar support area during immobilization compared to non-immobilization, regardless of the dominance of the immobilized limb (*p* < 0.001), with discernible differences between the dominant and non-dominant sides (Figure 2b).

Additionally, when disregarding immobilization and limb dominance, the impact of eye state (open vs. closed) on plantar support was evaluated. The results suggest that the eye state significantly influences plantar support, independent of limb immobilization or dominance (Table 3).

## 4. Discussion

Despite the importance of proprioception in long-term functional outcomes after wrist–hand immobilization [20], no previous studies have examined the effect that wrist or hand immobilization may have on plantar support and, consequently, on the disruptions in the development of daily activities. Our study aimed to investigate the impact of wrist immobilization on postural control, specifically examining antero-posterior oscillation and plantar pressure distribution, while also considering the roles of hand dominance and visual conditions. In line with our hypotheses, the results revealed that wrist immobilization, regardless of hand dominance, led to significant changes in both postural sway and plantar pressure parameters when compared to the non-immobilized condition. These findings suggest that even distal upper limb constraints can influence body balance.

There are several plantar pressure measurement systems, such as Footscan^®^ [21], TekScan^®^ [22], Emed^®^ [23], Pedar^®^ [24], WalkinSense^®^ [25], and Medicapteurs S-Plate^®^ [26], among others. There have been some foot-related research studies that have used the PodoPrint^®^ platform [27,28]. Reliability and repeatability of the PodoPrint^®^ system have been demonstrated, and normal values of plantar foot pressure have also been identified in a healthy population [16].

We already knew that the immobilization of the wrist or hand had consequences on the shoulder [29], and increased time of immobilization of the wrist was associated with an increase in shoulder pain in patients after wrist fracture. However, we were unaware of the potential effects that immobilizing the wrist or hand could have on plantar support, gait development, balance, and consequently, on the sensation of movement and agility during locomotion. The results of this study could have clinical implications in patients with conditions involving wrist immobilization, highlighting the importance of a global rehabilitation approach. Specifically, the findings elucidated on the relationship between wrist immobilization and various postural parameters revealed not only the dynamic interplay between upper limb function and lower limb biomechanics but also the influence of wrist–hand immobilization on these parameters [30]. Our results indicate that wrist immobilization alone induces changes in antero-posterior oscillation (APO), regardless of whether the immobilized hand is dominant or non-dominant and independent of visual conditions (eyes open or closed). However, when the eyes are closed, the length of APO increases, likely due to the involvement of the vestibular system [31].

Previous studies concluded that plantar pressure parameters can be modulated in response to different visual inputs [31,32]. Effects of visual stimuli on plantar pressure parameters were also reported in open-eye (OE) condition by Fullin et al. [33]. Similarly, P. De Blasiis et al. [34] investigated the impact of visual stimuli on postural stability and plantar pressure parameters in a healthy population, analyzing changes under both OE and closed-eye (CE) conditions. Their results confirm that the coefficient of variation was lower than 20% in CEs and OEs across all parameters, except for the right arch index, which suggests a greater dynamic role of the dominant foot with respect to the postural adaptations occurring with closed eyes. Also, Hébert-Losier, K et al. [33,35] concluded that plantar pressure parameters do not seem to be affected by the different visual conditions. These studies partially differ from our results, as we observed differences in participants depending on whether their eyes were open or closed. This discrepancy may be attributed to wrist immobilization, a variable not considered in previous studies. Our findings suggest that eye condition (open vs. closed) significantly impacts the distribution of plantar load, regardless of limb immobilization or dominance. Nevertheless, it is important to emphasize that irrespective of the eye condition or dominance, it is the immobilization itself that induces effects on both antero-posterior oscillation and the area of plantar support.

Upper extremity limitations impact gait, balance, and overall functionality [36,37]. This redistribution of plantar pressure may be indicative of an attempt to redistribute body weight in response to compromised upper limb function. Our study also revealed significant changes in plantar pressure distribution in individuals due to wrist immobilization. It is conceivable that individuals with wrist immobilization, either consciously or subconsciously, engage their lower limbs to a greater extent to compensate for reduced upper limb functionality during walking, as interlimb neural coupling influences muscle recruitment [7]. It is not the first time that some investigators reported the correlation between the hand and the foot; for example, Slobounov, S et al. reported referred differences between the parameters of hand strength and plantar pressure distribution [38,39]. Other studies that have focused on postural control have shown the right hemisphere’s pivotal role on balance control and body posture in healthy subjects [40,41]. In the same line, Rosario Emanuele Bonaventura et al. [42] investigated the effect of prismatic adaptation (PA) on handgrip strength and plantar pressure. They concluded that the PA effects on body posture are probably related to modulation of body representation.

Focus on body representation, and in concordance with our results, there are many results that converge on the conclusion that maintaining an upright posture requires the nervous system to integrate information from the somatosensory, visual, and vestibular systems. Therefore, any external factor that may influence this perception (such as wrist immobilization in our study) could impact the motor and sensory systems, leading to a series of mechanical and proprioceptive adjustments that are crucial for effective rehabilitation. These objective findings, which highlight the correlation between wrist immobilization and plantar support, could be the way for new research focused on clinical applications for upper limb injury rehabilitation.

However, our results are based on a healthy population; consequently, the impact of factors, such as kinesiophobia or post-injury wrist pain, on plantar support remains unknown. Moreover, a potential limitation of this study is the possibility of evaluator bias, as the evaluator was aware of the conditions being tested. Although efforts were made to ensure objective assessments, the lack of blinding may have introduced an unintended source of bias. Future research should address this limitation by implementing a double-blind design to enhance the reliability of the findings. Future research should integrate clinical assessments to strengthen the interpretation and clinical relevance of our findings. Additionally, further studies should explore the impact of various factors influencing plantar support following wrist immobilization, identifying both predictive and confounding variables, as well as potential changes occurring throughout the progression of the pathology.

## 5. Conclusions

Wrist–hand immobilization alone, independent of eye status (open or closed), resulted in significant alterations in antero-posterior oscillation and plantar pressure, with discernible differences between the dominant and non-dominant sides. The eye state significantly influenced plantar support, independent of limb immobilization or dominance.

## Figures and Tables

**Table 1:** Summary of the statistical significance of APO alterations due to immobilization alone.

	Value		
Predictors	Estimates	CI	*p*
(Intercept)	61.39	55.11–67.68	<0.001
Eyes [OE]	−9.51	−12.42–−6.60	<0.001
Dominant	−8.48	−12.05–−4.92	<0.001
Not dominant	−11.17	−14.74–−7.61	<0.001
Random effects			
σ^2^	134.31		
τ_00 DNI_	327.79		
ICC	0.71		
N_DNI_	41		
Observations	246		
Marginal R^2^/Conditional R^2^	0.090/0.735		

**Table 2:** Data on the impact of wrist and hand immobilization on total plantar support area.

	Value		
Predictors	Estimates	CI	*p*
(Intercept)	63.15	56.55–69.76	<0.001
Eyes [OE]	−13.03	−18.05–−8.01	<0.001
Dominant	−10.30	−15.32–−5.28	<0.001
Not dominant	−14.64	−19.66–−9.62	<0.001
Eyes [OE] x Dominant	3.64	−3.46–10.74	0.314
Eyes [OE] x Not dominant	6.93	−0.17–14.03	0.056
Random effects			
σ^2^	133.19		
τ_00 DNI_	327.97		
ICC	0.71		
N_DNI_	41		
Observations	246		
Marginal R^2^/Conditional R^2^	0.093/0.738		

**Table 3:** Exploring the effect of eye state on plantar support, disregarding immobilization and limb.

	Value		
Predictors	Estimates	CI	*p*
(Intercept)	27.60	20.37–34.82	<0.001
Eyes [OE]	−4.56	−8.88–−0.23	0.039
Dominant	−1.66	−6.95–3.63	0.537
Not dominant	−4.45	−9.74–0.84	0.099
Random effects			
σ^2^	296.04		
τ_00 DNI_	354.05		
ICC	0.54		
N_DNI_	41		
Observations	246		
Marginal R^2^/Conditional R^2^	0.013/0.551		

## Data Availability

The original contributions presented in this study are included in the article. Further inquiries can be directed to the corresponding author.
